# The impact of depressive and anxious symptoms on non-suicidal self-injury behavior in adolescents: a network analysis

**DOI:** 10.1186/s12888-024-05599-1

**Published:** 2024-03-26

**Authors:** Muzhen Guan, Junchang Liu, Xinhong Li, Min Cai, Jing Bi, Ping Zhou, Zhongheng Wang, Songwen Wu, Li Guo, Huaning Wang

**Affiliations:** 1 Department of Mental Health, Xi’an Medical College, 710021 Xi’an, China; 2 Department of psychiatry, the first affiliated hospital, Air Force Medical University, 710032 Xi’an, China; 3 Department of General Practice, Tangdu Hospital, Air Force Medical University, Xi’an, China; 4 Department of science, Xi’an Guanmiao primary school, 710086 Xi’an, China

**Keywords:** Non-suicidal self-injury, Adolescent; network analysis, Depressive symptoms, Anxious symptoms

## Abstract

**Purpose:**

Conceptualizing adolescent NSSI and emotional symptoms as a system of causal elements could provide valuable insights into the development of non-suicidal self-injury (NSSI) in adolescent. This study aimed to explore the intricate relationship between NSSI, depressive symptoms, and anxious symptoms in adolescents, identifying key symptoms to establish a theoretical foundation for targeted and effective interventions addressing NSSI behaviors in this population.

**Methods:**

A total of 412 adolescents with NSSI behaviors were selected from outpatients. Generalized anxious disorder scale (GAD-7) and patient health questionnaire (PHQ-9) were employed to measure anxious symptoms and depressive symptoms, respectively. The adolescent non-suicidal self-injury assessment questionnaire (ANSSIAQ) was used to evaluate NSSI of adolescent. Using network analysis, the NSSI、depressive symptoms and anxious symptoms network were constructed to identify the most central symptoms and the bridge symptoms within the networks.

**Results:**

The findings revealed that the NSSI functional nodes “coping with sadness and disappointment” and “relieving stress or anxious” exhibited the strongest correlation, with a regularized partial correlation coefficient was 0.401. The symptoms “having a desire to harm oneself and unable to stop” and the node “depressive symptoms” had the highest strength centrality in the network, and their strength centrality indices were 1.267 and 1.263, respectively. The bridge nodes were “having a desire to harm oneself and unable to stop” and “expressing one’s despair and hopelessness”, with expected impact indices of 0.389 and 0.396, respectively.

**Conclusion:**

In adolescents, the network revealed a closer connection between NSSI and depressive symptoms. “The desire to not stop hurting oneself” is not only broadly connected to other nodes but also could activate other nodes to maintain NSSI behavior. In light of these findings, precise targets for pharmacological treatment, psychotherapy, physical therapy, etc., are identified for adolescents with NSSI. Targeting this specific aspect in interventions may contribute to preventing and reducing NSSI behavior in adolescents.

## Introduction

Non-suicidal self-injury (NSSI) refers to a series of behaviors such as harming one’s body directly, repeatedly and intentionally without suicidal intent and in a way that would not lead to death [1]. The incidence of NSSI behavior in adolescents has increased dramatically. A systematic review study indicated a detection rate of 7.5–46.5% for NSSI behavior among adolescents [3], while Liu et al. surveyed a detection rate of 5.4–33.8% for NSSI among Chinese adolescents [4]. NSSI behaviors in adolescents often commence in early adolescence or even childhood [2], potentially persisting throughout adolescence and into adulthood. These behaviors directly impact the social functioning of adolescents, such as learning and interpersonal relationships. Studies have found that 70% of adolescents with NSSI behaviors reported engaging in suicidal behaviors, posing a direct threat to the life safety of adolescents [5]. Therefore, it directly threatens the life of adolescents and has become an increasingly serious health problem [6].

NSSI behavior among adolescents includes multiple methods, such as cutting, burning, scratching, head banging, and hitting [7]. Calvete et al. found that 32.2% of adolescents commit serious self-mutilation (such as cutting, burning, scratching) [8]. Adolescents often use multiple NSSI behaviors rather than one behavior [9]. Bildik et al. noted that there is a relationship between the frequency of different NSSI behaviors and the severity of physical injury, but inferring the severity of physical injury by directly calculating the frequency may produce misleading results [10]. For example, the severity of physical injury from cutting oneself 10 times is different than that of rubbing one’s skin 10 times.

The NSSI function has become a hot issue in current research [11]. The main functions of NSSI include emotional regulation, interpersonal regulation, sensory regulation, anti-dissociation and self-punishment [12, 13]. Among them, emotional regulation is one of the primary functions of NSSI in adolescents [14]. This theory holds that NSSI behavior is maintained by negative reinforcement [15]. Despite the long-term negative consequences, NSSI can reduce negative emotional states [16]. Studies have shown that depression may play aessential role in the development and persistence of NSSI [17, 18]. If individuals are stimulated externally and experiences severe depression, they may engage in self-injurious behaviors to alleviate depression [19]. In addition, a meta-analysis showed that depression had a significant effect on NSSI [20], and adolescents with NSSI behavior also suffered from depression [21, 22]. Previous studies have also observed a positive association between anxiety and NSSI [23, 24], and severe anxiety is considered to be a significant cause of NSSI [25]. A meta-analysis of 56 studies showed that patients with affective disorders, such as depression and anxiety, had a higher risk of NSSI behaviors [26].

In the network approach (NA), the observation variables are used as primary indicators, and the graph theory method is used to establish the relationship network between observation variables [27]. The variable is the node of the network, and the relationship between the variables is the line connecting the nodes [28]. Network analysis can highlight the relationship between the observational variables and the systems formed by the interaction of the observational variables [29]. In the network, nodes represent symptoms, and lines represent the interaction among symptoms [30]. Network analysis can provide corresponding centrality metrics for each node, including strength, closeness and betweenness. A node with high strength (or bridge-strength) may play a crucial role in activating or maintaining the entire network, and these metrics can offer potential targets for interventions [28].

In an increasing number of studies, NA has been used to explore the complex relationships among symptoms of mental disorders, including depression and severe depression with NSSI [31]. Mancinelli et al. found that low self-control and external problems were the key nodes of the adolescent NSSI symptom network through network analysis by selecting 155 adolescents with NSSI [32]. In addition, Hoorelbekea et al. explored the symptom network of adolescent depressive suicide and found that the core symptom of the depressive network was loneliness, which had the strongest association with suicidal ideation. Intervention in the core symptoms of loneliness can effectively reduce suicidal ideation [33].

Previously researches on non-suicidal self-injury (NSSI) in adolescents have primarily focused on studying its functions, behavioral patterns, and frequency. Studies have also explored the relationship between NSSI and depression and anxiety, often utilizing traditional statistical methods such as regression analysis or factor analysis. While these methods are effective in assessing the relationships between specific predictive variables and outcome variables, they fall short of capturing the interdependencies and complex interactions among multiple variables. This limitation becomes particularly pronounced when investigating complex phenomena like NSSI behaviors and emotions in adolescents.

Borsboom’s Network Theory of Mental Disorder (NTMD) suggests that the development and maintenance of mental disorders are influenced by dynamic causal relationships among various symptoms within the disorder. When these causal relationships are sufficiently robust, they form a feedback loop, perpetuating the disorder. According to NTMD, intervening in a mental disorder requires correcting the status of core symptoms within the network, triggering changes in the network’s structural configuration, and ultimately achieving therapeutic results [34]. The Network Analysis Method (NAM) aligns with the principles of NTMD. Recognized as a cutting-edge approach for analyzing psychiatric disorders, NAM demonstrates significant advantages in handling multi-variable behavioral data [35]. This method not only reveals the relationships among individual symptoms but also, through the network’s centrality metrics, facilitates the identification of core and bridge symptoms, providing a more comprehensive perspective on exploring the connection between NSSI behaviors and emotions in adolescents. Therefore, in this study, a network analysis method was used to construct a symptom network among NSSI, depressive and anxious symptoms in adolescents in order to explore how the mutual activation of symptoms can maintain NSSI behavior and to provide a starting point for interventions for NSSI behavior.

## Subjects and methods

### Subjects

Using a convenience sampling strategy, 412 untreated adolescent patients, aged 13–18 years and right-handed, were recruited from the psychiatry outpatient department of the First Affiliated Hospital of the Air Force Medical University from November 2022 to May 2023.

The inclusion criteria were as follows: patients who met the NSSI diagnostic criteria in the Diagnostic and Statistical Manual of Mental Diseases and were diagnosed by the psychiatrist according to the diagnostic criteria.

The exclusion criteria were as follows: ① those who suffered from severe physical diseases; ② those with history of brain trauma or brain surgery history; ③ those with a history of other mental or neurological disorders; ④ those with a high risk of suicide.

Before the experiment, the subjects read and understood the experimental procedures and precautions and signed the informed consent form. Then, psychiatrist provided a quick response code, and participants answered the questionnaire by scanning the code with their mobile phones. This study was approved by the Ethics Committee of the First Affiliated Hospital of the Air Force Medical University (20,222,058-F-1).

### Methods

#### General information questionnaire

This study utilized a self-designed general information questionnaire to collect personal information from adolescents, including age, gender, years of education, parental contact details, etc.

#### Generalized anxiety disorder scale

The Generalized anxiety disorder scale (GAD-7) is a self-rating scale for assessing anxious symptoms with 7 entries. It has good reliability and validity in assistant diagnosis and symptom severity assessment of anxious disorders. The internal consistency of the GAD-7 was excellent (Cronbach α = 0.92). Test-retest reliability was also good (intraclass correlation = 0.83) [36].

#### Patients health questionnaire

The patient health questionnaire (PHQ-9) is a simple and effective self-rating scale for depressive symptoms with 9 entries. It has good reliability and validity in the assisted diagnosis and symptom severity assessment of depressive symptoms. The PHQ-9 exhibits excellent a good Cronbach α consistency (Cronbach α = 0.87) [37].

#### Adolescent non-suicidal self-injury assessment questionnaire

The adolescent non-suicidal self-injury assessment questionnaire (ANSSIAQ) includes two subscales, namely, the behavior questionnaire and the function questionnaire. There were 19 items in the function questionnaire. There were 12 items in the behavior questionnaire, which was further divided into two factors: (1) self-injury behavior without obvious tissue injury, which referred self-injury behavior that did not cause obvious and serious body tissue injury, such as pinching, scratching, and hair-pulling; and (2) self-injury behavior with obvious tissue injury, which referred to self-injury behavior that may result in massive bleeding, scratches and other tissue damage, such as cutting, burning, lettering or writing symbols on the skin. The ANSSIAQ demonstrates robust reliability and validity, with a Cronbach’s α coefficient of 0.921 for the behavioral questionnaire, split-half reliability of 0.851, and test-retest reliability of 0.843; additionally, the functional questionnaire has a Cronbach’s α coefficient of 0.905, split-half reliability of 0.786, and test-retest reliability of 0.805 [38].

#### Statistical analysis

Statistical analysis was conducted using SPSS23.0 to calculate the GAD-7 and PHQ-9 total scores and the average and standard deviation of the each item in NSSI behavior questionnaire and function questionnaire.

The Gauss graph model (GGM) was used to fit the data, and R software was used for statistical analysis and visualization of the regularization partial correlation network. The GGM is a model used for analyzing relationships between variables, particularly well-suited for handling complex symptom relationships [39]. A more stable and easy-to-interpret regularization partial correlation network is obtained by using a graphical Lasso algorithm [40]. The network is displayed according to the layout of the Fruchterman-Reingold (FR) algorithm. The nodes with strong connections are located in the center of the network, while the nodes with weak connections are located in the periphery of the network [41]. A blue line in the network indicates a positive correlation, and a red line indicates a negative correlation. A thicker edge indicates that the correlation between two nodes is greater, and a thinner edge indicates that the correlation between two nodes is weaker.

The centrality index, including strength centrality (SC), closeness centrality (CC), and betweenness centrality (BC) [42], was calculated using the R package qgraph. The strength centrality of a symptom is equal to the absolute sum of the weights of the edges connected to the symptom. Closeness centrality indicates the average distance between a symptom and all other symptoms in the network. Betweenness centrality is equal to the number of times that a symptom is located on the shortest path length between any two other symptoms [43]. Strength centrality may be the most important and meaningful centrality index, and it is more likely to affect the entire network when nodes with high strength centrality are activated [28].

In addition, the bridging centrality index is calculated, including the bridge strength, bridge closeness and bridge betweenness. Among them, bridge strength is the best indicator to identify bridging nodes. If the bridge node is disconnected from the surrounding nodes, it can prevent the spread of symptoms [44]. The bridge expected influence is calculated through the sample-based bootstrap method (1000 bootstrap samples) using the R-package bootnet [45].

The accuracy and stability of the network were assessed using the bootnet package. Firstly, non-parametric bootstrapping (1000 bootstrap samples) was employed to compute the 95% confidence intervals (CI) for the evaluation of edge weight accuracy. Secondly, sample drop bootstrapping (1000 bootstrap samples) was utilized to calculate the correlation stability (CS) for assessing the stability of expected node influences and bridge expected influences. Previous research suggests that the CS value is ideally above 0.50 and should not fall below 0.25.

## Results

### Results of general description of the subjects

A total of 436 questionnaires were distributed and collected in this study, and after excluding data with missing values or outliers, the effective questionnaires were 412, effective rate was 94.49%. The age of the subjects ranged from 13 to 18 years old, and the proportion of females was 70.15%. Table 1 shows the demographic data of the subjects and the GAD-7, PHQ-9 and NSSI scores.

**Table 1:** Analysis of general description of the subjects

	Mean ± SD
Age	15.034 ± 1.635
M/F	123/289
Education (year)	7.104 ± 1.365
GAD-7	13.301 ± 5.043
PHQ-9	18.575 ± 6.175
NSSI behavior	16.158 ± 11.932
Self-injury behavior without obvious tissue injury (B1)	9.144 ± 7.474
Self-injury behavior with obvious tissue (B2)	7.014 ± 5.065
NSSI function	36.712 ± 10.826

Note: NSSI: GAD-7: generalized anxiety disorder-7; PHQ-9: patient health questionnaire-9

### NSSI functional scale score results

The score results of each item on the NSSI functional scale are shown in Table 2.

**Table 2:** Each item scores of the NSSI functional scale

Items	mean ± SD
F1. Express anger	3.274 ± 1.142
F2. To avoid things that I do not like or makes myself unhappy (such as going to school, doing homework, or working)	3.206 ± 1.270
F3. Make myself feel less lonely	2.767 ± 1.209
F4. Relieve stress or anxiety	3.890 ± 1.121
F5. Ways of self-punishment	3.308 ± 1.300
F6. Can bring happiness, enjoyment, and make myself feel good	2.904 ± 1.315
F7. Control myself to calm down	3.507 ± 1.222
F8. Attracting others’ attention	2.144 ± 1.108
F9. Revenge others	1.911 ± 1.043
F10. Only in this way can I avoid harming others	3.048 ± 1.430
F11. Protect myself from others’ attacks	2.322 ± 1.237
F12. help myself stop negative thoughts	3.220 ± 1.295
F13. My friends have done this before	2.027 ± 1.095
F14. Having a desire to harm myself and cannot stop	3.404 ± 1.235
F15. Gaining others’ understanding	2.329 ± 1.145
F16. Coping with sadness and disappointment	3.815 ± 1.024
F17. Expressing my despair and hopelessness	3.315 ± 1.264
F18. Letting others make changes	2.363 ± 1.150
F19. Escaping from numbness and illusion	2.959 ± 1.264

### Network analysis results

**Fig. 1:**
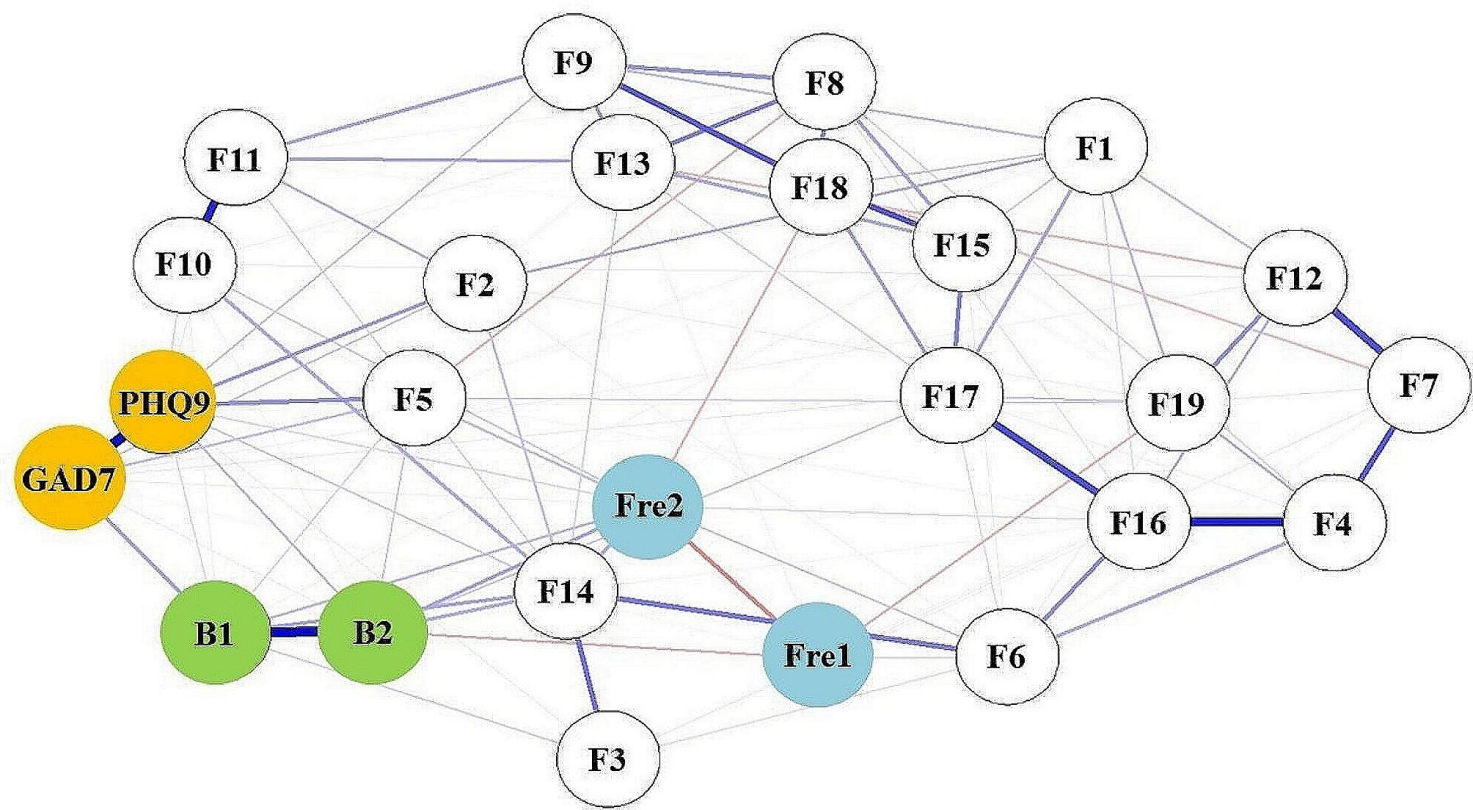
Network structure of NSSI with depressive and anxious symptoms in adolescents.

The regularized partial correlation network of NSSI, depressive and anxious symptoms is shown in Fig. 1. Blue lines indicate a positive correlation, and red lines indicate a negative correlation. Blue edges represent positive correlations, red edges represent negative correlations. The thicker edge reflects the magnitude correlation. Five groups of symptoms in the items of the functional subscale show obvious regularized partial correlation. They are “coping with sadness and disappointment (F16)” and “relieving stress or anxiety (F4)”, “only in this way can I avoid harming others (F10)” and “protect myself from others’ attacks (F11)”, " Expressing my despair and hopelessness (F17)” and “coping with sadness and disappointment (F16)”, " Control myself to calm down (F7) " and " help myself stop negative thoughts (F12)”, and “relieving stress or anxiety (F4)” and “controlling myself to calm down (F7)”, with corresponding regularized partial correlation coefficients of 0.404, 0.403, 0.402, 0.401 and 0.400, respectively.

### Centrality analysis results

**Fig. 2:**
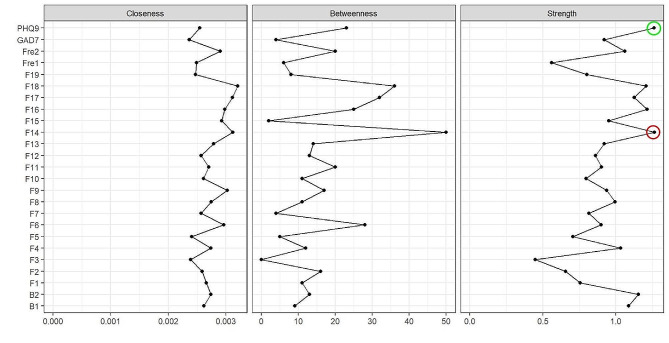
Centrality plot of the regularized partial correlation network

The centrality index (Z score) of NSSI symptoms with depressive and anxious symptoms is shown in Fig. 2. ȌHaving a desire to harm myself and cannot stop (F14)” and “depression (PHQ-9)” have the strongest strength centrality, with indices of 1.267 and 1.263, respectively. This indicates that these two nodes are the most important in the network and have the widest association with other nodes, altering these two nodes would lead to changes in other nodes within the network.

**Fig. 3:**
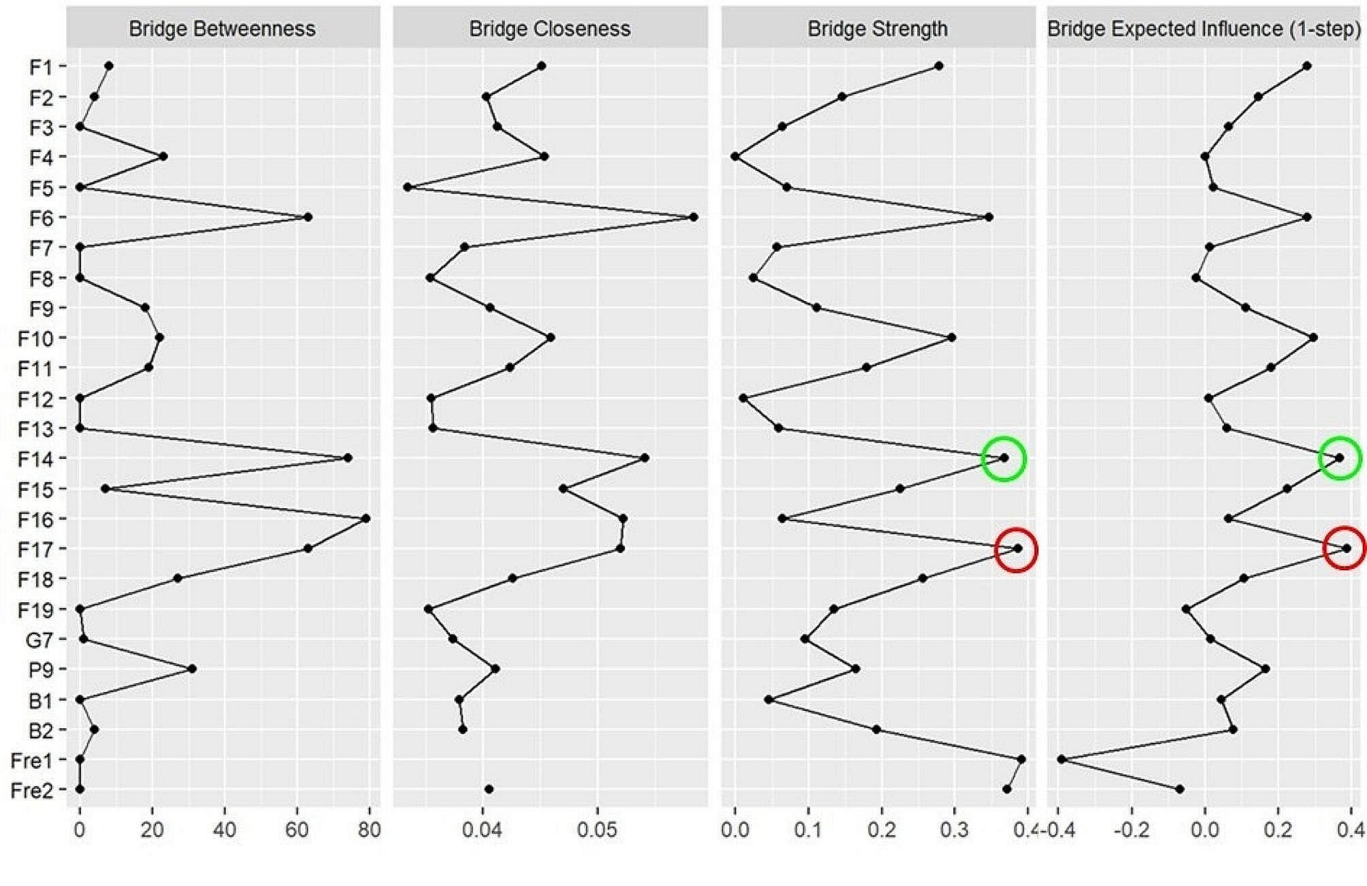
Bridge centrality plot of NSSI with depressive and anxious symptoms network in adolescent

The larger value of the Bridge Expected Influence, the more it represents a bridge symptom in the network. In network, the bridge strength and the bridge expected influence indicate that " Having a desire to harm myself and cannot stop (F14)” and “expressing my despair and hopelessness (F17)” are bridge symptoms (Fig. 3). Among them, the bridge strength of F14 is 0.365, and the expected influence is 0.389; the bridge strength of F17 is 0.388, and the expected influence is 0.396. Nodes with high Bridge strength and Bridge expected influence can activate other nodes, spreading throughout the entire network. They are key nodes in the maintenance and development of NSSI.

### Estimation of network accuracy and stability

**Fig. 4:**
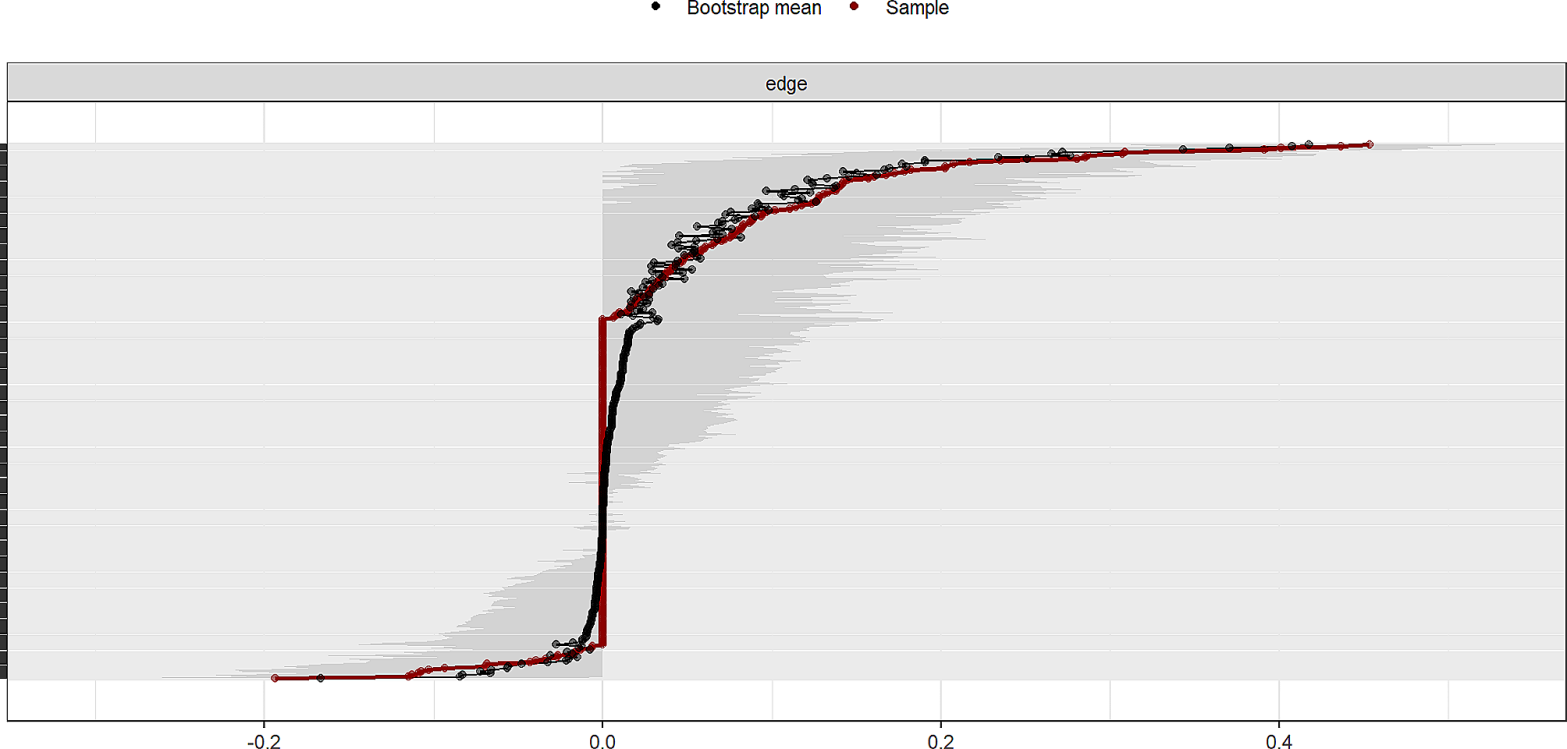
Results of the accuracy of edge weights

Figure 4 displays the accuracy of the bootstrap method in obtaining edge weights. The narrow confidence interval range indicates that the edge weights have sufficient accuracy.

**Fig. 5:**
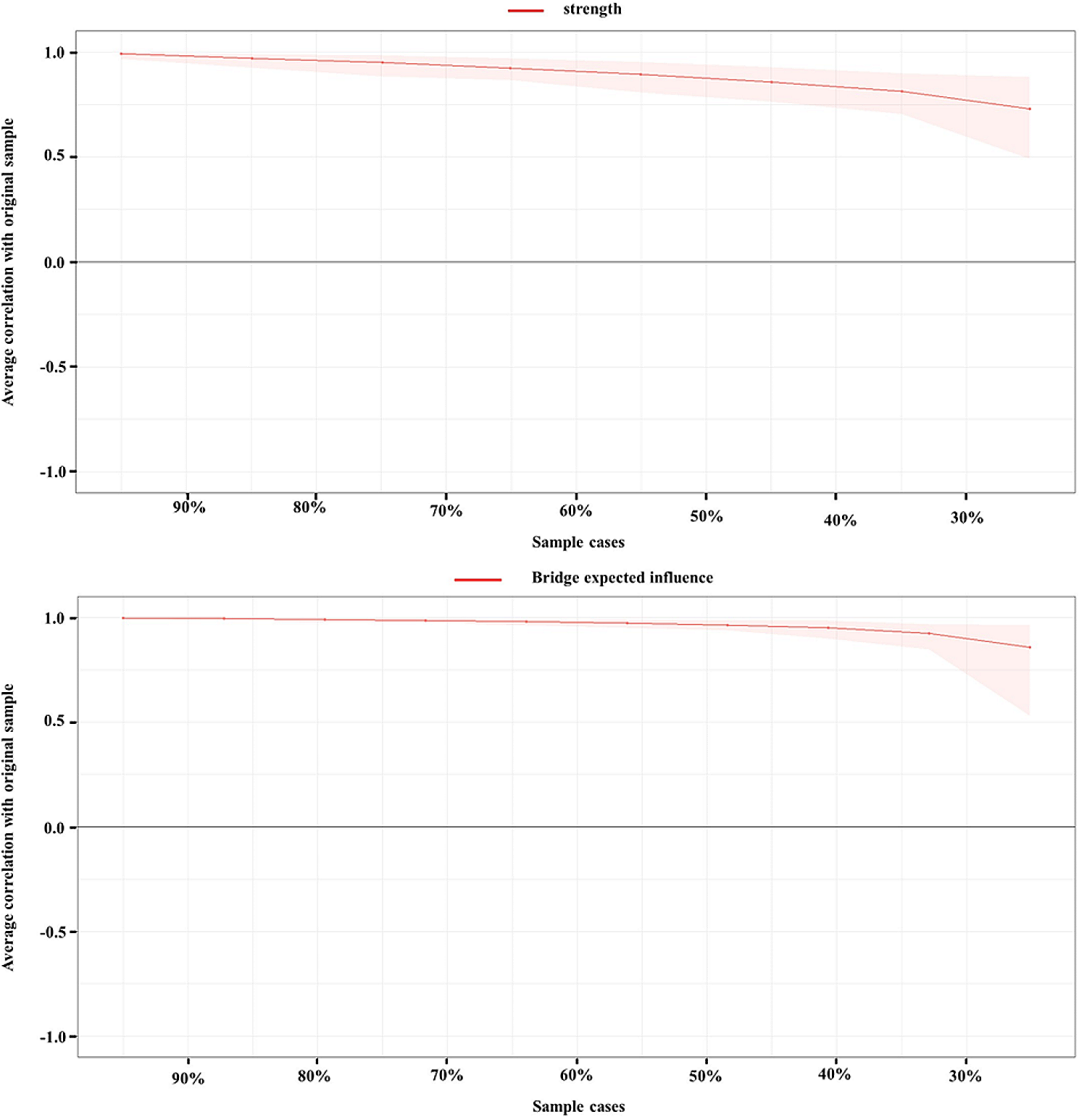
The stability of network structure by case-dropping bootstrap. Note: **A** depicts the stability of strength centrality; **B** shows the stability of bridge expected influence

Figure 5 displays the stability coefficients for strength centrality and bridge expected influences. With coefficients at 0.75 for both, it signifies adequate stability in strength centrality and bridge expected influences.

## Discussion

In this study, depressive symptoms, anxious symptoms and NSSI behaciors were used as nodes to construct the regularized partial correlation network of adolescents with NSSI. The results indicate that the strongest connection lies between “Coping with sadness and disappointment (F16)” and “relieving stress or anxiety (F4)”, “Expressing my despair and hopelessness (F17)” and “controlling myself to calm down (F7)”. “Having a desire to harm myself and cannot stop (F14)” and “depression (PHQ-9)” are the central symptoms of the network. The bridge nodes in network are " Having a desire to harm myself and cannot stop (F14)” and “expressing my despair and hopelessness (F17)”.

The highly connected nodes in the regularized partial correlation network are mainly the entries in the NSSI functional scale. The stronger connections are between F16-F4, F10-F11, F17-F16, F7-F12, and F4-F7. Most of these nodes involve items “Relieve stress or anxiety (F4)”, “Control myself to calm down (F7)”, “Coping with sadness and disappointment (F16)”, and “Expressing my despair and hopelessness (F17)”, which are related to coping with negative emotions, while “help myself stop negative thoughts (F12)” is associated with negative thoughts. Negative thoughts often coexist with negative emotions. Therefore, the primary function of adolescent NSSI behavior is to relieve feelings of despair, sadness, hopelessness, and other negative emotions. Adolescence is a period of increased emotional response in adolescents, and despair and hopelessness play an important role in the occurrence of emotional disorders in adolescents [46]. When adolescents feel hopeless, they can express negative emotions such as despair through NSSI. In addition, as an emotional regulation strategy, NSSI regulates negative emotions and can alleviate depressive or anxious symptoms. This is consistent with NSSI’s theory of emotional regulation [47].

From the centrality analysis results, it can be seen that “Having a desire to harm myself and cannot stop (F14)” is both the central node and the bridge node of the network. This node has the most extensive and close connection with other nodes in the network. As the central node of the network, this node is also connected to the NSSI behavior mode, frequency of occurrence, and time of the last occurrence. In addition, as a bridge node, once activated, it activates other nodes through the edges connecting other nodes and propagating throughout the entire network [31]. From the perspective of the entire network, the activation of this node will have a positive effect in regulating negative emotion and promoting the NSSI behavior function. However, it will also exacerbate depression and even induce self-injury behavior that clearly harms oneself, and it can also increase the frequency of self-injury. “Having a desire to harm myself and cannot stop” (F14) reflects the weak control of adolescents over NSSI behavior. From the perspective of neurodevelopment, adolescent NSSI behavior stems from the interaction between the fragility of the developing nervous system and stressors [48]. When perception of stress increases in adolescent [49], there is an imbalance in functional transitions between the prefrontal cortex regions that regulates emotions and impulses from top to down, and the subcortical region involved in emotional experience generation and reward seeking, which results in low self-control in adolescents, leading to engagement in NSSI behaviors [50].

After entering adolescence, there is an increased prevalence of NSSI behaviors among adolescents [51], the risk of depression increases [52], and NSSI often occurs simultaneously with depressive symptoms [53, 54]. PHQ-9 reflects depressive symptoms and is another central node in the network. This node is also connected to the NSSI functional node and the F14 node. The aggravation of depression causes adolescents to uncontrollably harm themselves, thereby maintaining the occurrence of NSSI behaviors. However, in this study, the results did not show anxious symptoms to play a central role in the network, which is possibly ascribed to the fact that NSSI behavior itself is mainly used to alleviate depression rather than anxiety [55]. NSSI and depression might emerge together as a result of common genetic, cognitive, or social risk factors [56]. A meta-analysis examining risk factors for adolescent NSSI highlighted depression as a prominent contributing factor [57]. Wu et al. proposed a mediation model, suggesting that difficulty in emotion regulation (DER) and depression mediate the relationships between behavioral inhibition/approach systems (BIS/BAS) and NSSI. Adolescents with higher levels of BIS or with lower levels of BAS are more likely to encounter DER, characterized by heightened negative emotional reactivity and maladaptive efforts to regulate intense emotions. As individuals struggle to adaptively manage negative emotions and resort to suppression or avoidance, there is a gradual accumulation, leading to heightened negative emotional experiences. This, in turn, may contribute to elevated levels of depression, ultimately increasing possibility of NSSI [58].

“Expressing my despair and hopelessness (F17)” is another bridge node in the network that would be able to activate other nodes connected to it. From the network, it can be seen that this node is mainly closely connected to other NSSI functional nodes, and its activation can be transmitted to other NSSI functional nodes. It can cope with emotions such as sadness and disappointment. The pain caused by hurting can help adolescents escape from numbness and illusion. It can also gain the understanding of others and allow them to make changes [59]. A study found that feelings of despair and hopelessness in adolescents lead to more loneliness and depression [60, 61], which may trigger the occurrence of self-injury behavior [62]. The research findings indicate that with the persistence of the hopeless feeling, individuals may develop a desperate perception of themselves and even of the whole world. At this point, NSSI may be utilized as an effective strategy to alleviate despair and hopelessness. Hopelessness serves as a crucial mediating factor for NSSI; addressing hopelessness can effectively reduce NSSI [63]. Psychological therapy is a crucial method for regulating feelings of hopelessness, guiding adolescents to explore positive resources within themselves and their surroundings, and helping them discover that assistance is available [64]..

Our study has some limitations. Firstly, it is cross-sectional and lacks a longitudinal design. Future research will involve regular tracking and data collection at different time points, allowing for a more comprehensive temporal analysis. This approach aims to delve deeper into the changes in adolescent NSSI over different periods, providing a foundation for understanding NSSI. Secondly, although the network of adolescent NSSI constructed in this study involves various variables, NSSI is related to many factors. In future research, we plan to incorporate more study variables, such as gender, age, parenting styles, etc. Lastly, this study is a behavioral investigation into adolescent NSSI. Despite employing network analysis, our understanding of the mechanisms underlying adolescent NSSI is limited. Therefore, in future studies, we intend to explore the mechanisms of adolescent NSSI using techniques such as eye movement tracking, event-related potentials, functional magnetic resonance imaging, and other advanced technologies.

NSSI behavior is a clinical manifestation frequently observed in adolescents with mental disorders. These individuals not only exhibit NSSI behaviors but may also experience symptoms of depression and anxiety. Therefore, rapid relief of NSSI, a high-risk behavior, is crucial when formulating treatment plans. According to the conclusions of this study, when selecting medications, attention should be given to the antidepressant effects of the chosen drugs. In psychotherapy, adolescents with NSSI may describe instances of engaging in NSSI behaviors before exams or during conflicts with peers or parents. In these situations of exam anxiety, interpersonal stress, etc., underlying depressive emotions such as feelings of hopelessness, worthlessness, and desperation may be concealed. The study emphasizes the closer association between depressive symptoms、hopelessness and NSSI, guiding psychotherapists to delve into the potential depressive emotions and hopelessness behind these anxieties rather than addressing anxiety directly. In rTMS therapy, the choice of treatment targets should also consider targets with antidepressant effects.

This study explored the organization of NSSI behavior, depressive symptoms, and anxious symptoms in the network and preliminarily constructed a relationship network among NSSI behavior, depressive symptoms, and anxious symptoms in adolescents. This study found that NSSI and depression are closely related in the network structure in adolescents. The NSSI functional node “Having a desire to harm myself and cannot stop (F14)” is widely connected to other nodes and can activate them to spread to the entire network [65, 66]. By intervening on this node, it may be possible to effectively block the spread of symptoms and prevent the maintenance of NSSI behavior. The current study provide theoretical guidance for interventions in NSSI behavior in adolescents.

## Data Availability

Data are available from the corresponding author upon reasonable request.

## Abbreviations

NSSI: Non-suicidal self-injury
NA: Network approach
GAD-7: Generalized anxiety disorder scale
PHQ-9: Patient health questionnaire
ANSSIAQ: Adolescent non-suicidal self-injury assessment questionnaire
GGM: Gauss graph model
FR: Fruchterman-Reingold
SC: Strength centrality
CC: Closeness centrality
BC: Betweenness centrality
